# Thromboprophylaxis during the Pregnancy-Puerperal Cycle - Literature Review

**DOI:** 10.1055/s-0040-1708096

**Published:** 2020-04

**Authors:** Suzanna Maria Viana Sanches, Monique Magnavita Borba da Fonseca Cerqueira, Patrícia Lima Junqueira, Miriam Takayanagi Gomez

**Affiliations:** 1 Santa Casa da Bahia, Salvador, BA, Brazil; 2 Universidade do Estado da Bahia, Salvador, BA, Brazil; 3 Universidade Federal da Bahia, Salvador, BA, Brasil; 4 Sociedade Brasileira de Angiologia e Cirurgia Vascular, Salvador, BA, Brazil

**Keywords:** thromboembolism, thrombosis, pregnancy, postpartum, disease prevention, Tromboembolismo, trombose, gestação, pós-parto, prevenção de doenças

## Abstract

**Objective** To identify current strategies and recommendations for venous thromboembolism prophylaxis associated with the pregnancy-puerperal cycle, a condition of high morbidity and mortality among women.

**Methods** The literature search was performed between May and October 2019, using the PubMed database, including papers published in Portuguese, English and Spanish. The terms *thromboembolism* (Mesh) AND *pregnancy* (Mesh) OR *postpartum* (Mesh) were used as descriptors, including randomized controlled trials, meta-analyses, systematic reviews and guidelines published from 2009 to 2019, presenting strategies for prevention of thromboembolism during pregnancy and the postpartum.

**Results** Eight articles met the inclusion criteria. Many studies evaluated were excluded because they did not address prevention strategies. We compiled the recommendations from the American Society of Hematologists, the American College of Obstetricians and Gynecologists, the Royal College of Obstetricians and Gynecologists, the Society of Obstetricians and Gynaecologists of Canada, the American College of Chest Physicians and the Royal Australian and New Zealand College of Obstetricians and Gynaecologists.

**Conclusion:** There are some gaps in the research, and clinical studies with appropriate methodology are needed to support decisions made regarding the risk of thromboembolism in the perigestational period. Thus, the attention of the professionals involved in the care of pregnant and postpartum women is crucial, as it is a condition associated with high morbidity and mortality.

## Resumo

**Objetivo** Identificar as estratégias e recomendações atuais para profilaxia de tromboembolismo venoso associado ao ciclo gravídico-puerperal, condição de alta morbimortalidade entre mulheres.

**Métodos** A busca na literatura ocorreu entre maio e outubro de 2019, com pesquisa na base de dados do PubMed, contemplando trabalhos publicados nos idiomas português, inglês e espanhol. Os termos *thromboembolism* (Mesh) AND *pregnancy* (Mesh) OR *postpartum* (Mesh) foram utilizados como descritores, incluindo ensaios clínicos randomizados, metanálises, revisões sistemáticas e diretrizes publicadas entre 2009 a 20019, apresentando estratégias de prevenção de tromboembolismo venoso durante a gravidez e o pós-parto.

**Resultados** Oito artigos abordando estratégias de tromboprofilaxia primária e secundária durante a gestação, parto e puerpério foram selecionados para a presente revisão sistemática. Muitos estudos avaliados foram excluídos por não abordarem estratégias de prevenção. Foram compiladas as recomendações das seguintes sociedades: American Society of Hematologists, American College of Obstetricians and Gynecologists, Royal College of Obstetricians and Gynecologists, Society of Obstetricians and Gynaecologists of Canada, American College of Chest Physicians e Royal Australian and New Zealand College of Obstetricians and Gynaecologists.

**Conclusão** Até o presente momento, há algumas lacunas e estudos clínicos com metodologia adequada se fazem necessários para respaldar a tomada de decisão frente ao risco de tromboembolismo venoso no período perigestacional. Torna-se fundamental a atenção dos profissionais envolvidos no atendimento às gestantes e puérperas, pois trata-se de uma condição associada a alta morbimortalidade.

## Introduction

Venous thromboembolism (VTE), manifested as pulmonary embolism (PE) or deep-vein thrombosis (DVT), affects ∼ 1 to 2 per 1,000 pregnancies. Despite its low-incidence rates, it stands out as a relevant etiology of maternal morbimortality as it is the cause of 10 to 15% of deaths occurring during the pregnancy-puerperal cycle.^1^ ^2^ However, it is a preventable condition if adequate measures for thromboprophylaxis are provided.

Pregnant women present a four times greater risk of presenting with VTE when compared to nonpregnant women in the same age group, and the occurrence of DVT is more common, especially in the left lower limb, throughout pregnancy.^3^ During the postpartum period, the increase in risk is approximately 10 times greater,^4^ with PE being the most frequent manifestation when compared to isolated DVT.

The risk of VTE exists beginning in the first 3 months of pregnancy, before anatomical alterations become visible.^5^ It persists during the whole pregnancy, increases in the 3^rd^ trimester, and markedly rises during the postpartum.^6^ ^7^ Statistically, the number of incidences during pregnancy is similar to the puerperium, but considering the shorter duration of the postpartum, the daily risk is higher during the first weeks after giving birth, especially the first 7 days, when 50% of such events occur.^8^

Callaghan et al^9^ reported a 72% increase in the incidence of VTE in women admitted for childbirth between the years of 1998 and 2009, attributed to an increase in the prevalence of prothrombotic conditions such as obesity, maternal age, cesareans and other comorbidities.

Recently, epidemiological data has also evidenced a substantial growth in the incidence of VTE during recent decades. When assessing hospitalizations due to VTE during the pregnancy-puerperal cycle, it is possible to verify an estimated increase during pregnancy and the puerperium of 17% and 47%, respectively,^10^ reinforcing the need for adoption of specific measures for thromboprophylaxis.

Morbidity due to VTE during pregnancy can be acute or delayed, with significant impact on the quality of life of the patient.^11^ Pulmonary hypertension occurs in ∼ 4% of patients within 2 years of a PE diagnosis. The occurrence of post-thrombotic syndrome (PTS) was observed in 42% of women with DVT and in 24% of women who presented PE related to pregnancy.^12^

Thromboembolic disease imposes risks both to the mother and the fetus, and the peculiarities intrinsic to the pregnancy-postpartum period make thromboprophylaxis challenging in this context.

The available guidelines for thromboprophylaxis during pregnancy and the postpartum present a certain degree of uncertainty due to the lack of studies performed on this specific population, sometimes resulting in extrapolation based on data from studies that examined nonpregnant patients.

The Royal College of Obstetricians and Gynaecologists (RCOG),^13^ the American Society of Hematologists (ASH),^14^ and the American College of Obstetricians and Gynaecologists (ACOG)^8^ ^15^ published new guidelines with recommendations that were both accordant and incongruent with previous publications. Before that, recommendations were published by the Society of Obstetricians and Gynaecologists of Canada (SOGC)^16^ in 2014, the American College of Chest Physicians (ACCP)^17^ in 2012, and by The Royal Australian and New Zealand College of Obstetricians and Gynaecologists (RANZCOG)^18^ in 2012.

The aim of the present study is to identify strategies and recommendations for primary and secondary prophylaxis during pregnancy, childbirth and the puerperium, according to current knowledge, describing the complexity and relevance of the subject, which often makes valuable the conjoint approach of obstetricians, vascular surgeons and hematologists. For this purpose, a review will be made about the pathophysiological aspects of VTE in the pregnancy-puerperal cycle and the recommendations of the main guidelines identified will be compiled. It should be emphasized that guidance specifically concerning thrombophilia is outside the scope of the present article, and a consultation to the guidelines recommended by the ASH^14^ and the ACOG,^8^ both published in 2018, is advised.

## Methods

**Fig. 1:**
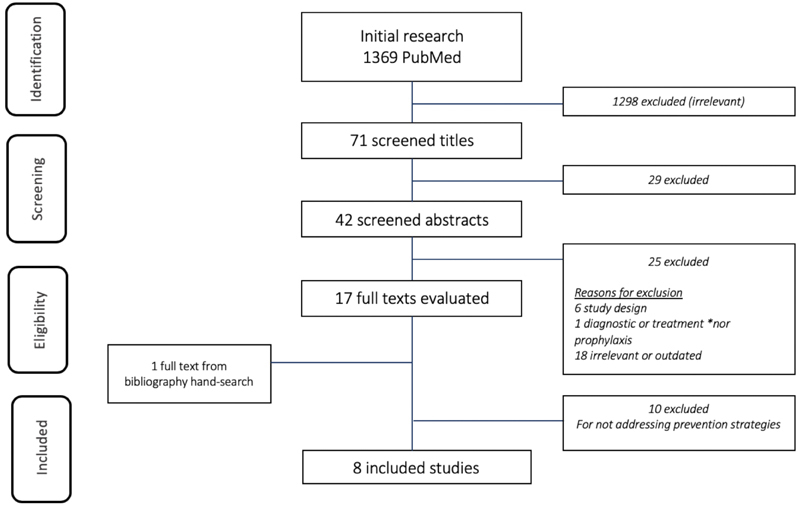
Flowchart of article eligibility and final inclusion in the present systematic review. **Source:** Reducing the risk of venous thrombosis and embolism during pregnancy and the puerperium.^13^

The literature review for the proposed research was conducted between May and October of 2019 using an online search in the PubMed – U.S. National Library of Medicine Databases. The terms *thromboembolism* (Mesh) AND *pregnancy* (Mesh) OR *postpartum* (Mesh) were used as descriptors. The search included randomized controlled trials, meta-analyses, systematic reviews and guidelines published from 2009 to 2019, presenting strategies for prevention of VTE during pregnancy and the postpartum. Abstracts that met the inclusion criteria and were published in Portuguese, English and Spanish were considered. Studies performed in populations outside the pregnancy-puerperal cycle, with specific thrombophilia or with thrombosis in atypical sites were excluded. “Grey literature” (unpublished) has been identified by searching the websites of databases as well as national and international medical societies. The studies were selected by title and then by summary by the same authors who conducted the research. The papers that met the eligibility criteria were fully evaluated. One relevant full text – The RCOG guideline^13^–from a bibliography hand search was included for its relevance. The study inclusion process is shown in Figure 1.

## Results

Of the articles selected from the database and those manually included, eight met the inclusion criteria. Many studies evaluated were excluded because they did not address prevention strategies. One randomized controlled trial evaluated two doses of enoxaparin specifically in obese women. The other studies included were guidelines from medical societies. Information was analyzed regarding authorship, year of publication, study design and main findings (Box 1).

**Table 1:** Thromboprophylaxis regimen with low molecular-weight heparin doses calculated by weight (adapted from the Royal College of Obstetricians and Gynaecologists)^13^

Weight	Enoxaparin	Dalteparin
< 50Kg	20mg/day	2500 U/day
50–90 Kg	40 mg/day	5000 U/day
91–130 Kg	60mg/day	7500 U/day
131–170 Kg	80 mg/day	10,000 U/day
> 170 Kg	0,6mg/Kg/day	75 U/Kg/day
High prophylactic dosage for women between 50–90Kg	40mg 12/12h	5,000 U 12/12h

**Source:** Reducing the risk of venous thrombosis and embolism during pregnancy and the puerperium.^13^

## Physiopathology of Venous Thromboembolism during Pregnancy, Labor and the Postpartum

Pregnancy is a prothrombotic state due to physiological and anatomical alterations that comprise the three key elements of the Virchow triad: venous stasis, hypercoagulability and endothelial lesion. Stasis is due to the compression of the pelvic vessels and the inferior vena cava by the pregnant uterus.^3^ ^20^ The physiological increase of coagulating molecules such as fibrinogen, VII, VIII, X and von Willebrand factors, PAI 1 and 2 and the concomitant reduced synthesis of the natural anticoagulant S protein lead to a hypercoagulable state.^21^ These alterations presumably represent an evolutional gain, with the purpose of reducing hemorrhagic complications, mainly during the peripartum and the puerperium. Endothelial injury is a consequence of vascular damage during labor and childbirth (vaginal or cesarean section).

## Risk Factors for Thromboembolism in the Pregnancy-Puerperal Cycle

It is possible to divide the risk factors for VTE as preexisting, obstetric and transitory.^13^ Among preexisting factors, there is prior occurrence of VTE; maternal age > 35 years old; body mass index (BMI) > 30 before pregnancy or during early pregnancy; multiple births (> 3 children); hereditary thrombophilia (antithrombin deficiency, protein C deficiency and protein S deficiency, mutation in the prothrombin gene and presence of the factor V Leiden); antiphospholipid syndrome; presence of comorbidities (cancer, heart failure, autoimmune diseases, intestinal inflammatory diseases, nephrotic syndrome, diabetic nephropathy); smoking; large varicose veins (symptomatic or above the knee or associated to phlebitis, edema or trophic alterations) and reduced mobility (i.e.: paraplegia).^13^

From the obstetric viewpoint, the risk conditions are multiple pregnancies; present pre-eclampsia; cesarean section, with four times greater risk than vaginal delivery; prolonged labor, with a duration of > 24 hours; use of rotation forceps; premature delivery or fetal death and postpartum hemorrhage, considered when there is a loss of > 1 liter of blood or when a blood transfusion is required.^13^

Other factors to be considered are surgical procedures during pregnancy or the puerperium (with the exception of perineal raphe); dehydration due to hyperemesis; ovarian hyperstimulation syndrome (only in the 1^st^ trimester); assisted reproduction and in vitro fertilization (IVF); hospitalization or immobility (> 3 days in bed); presence of systemic infection and long-distance travel (duration of > 4 hours).^13^

## Thromboprophylaxis Methods during Pregnancy

Considering the risks of the mother-baby binomial and possible morbidities that may occur after a thromboembolic event, the implementation of thromboprophylaxis measures are mandatory in all health institutions rendering obstetric care. The prevention of thrombus formation can be achieved by mechanical or pharmaceutical methods.^22^

## Mechanical Methods

Mechanical methods, which include deambulation, elastic compression and pneumatic compression, regulate the stasis risk factor by increasing the venous flow without elevating the risk of bleeding. These methods have been efficient in reducing the risk of VTE in two thirds of the general surgical population. Considering that data is scarce for the use of mechanical methods during pregnancy, the benefits are based on the results found in the surgical population outside the pregnancy-puerperal cycle.^22^ The medical team must assure to maintain the pregnant patient as active as possible and assess the risks and benefits of pharmaceutical thromboprophylaxis in situations of reduced mobility (such as hospitalization or prolonged bed rest). Elastic stockings, indicated as adjuvants in the prophylaxis of obstetric VTE, have been efficient in promoting an increase in venous flow and a significant reduction in the caliber of the femoral vein, reducing venous stasis. Although they are commonly prescribed for women who have recently given birth, their use is possible throughout all phases of pregnancy.^23^ ^24^ There are various models of sequential compression devices and there is no evidence of superiority among them in the prevention of VTE prophylaxis.^25^

## Pharmacological Methods

The benefits of anticoagulants in the prevention of VTE need to be confronted with the increase of hemorrhagic risk during pregnancy, the peripartum and the puerperium. There is heterogeneity amongst guideline recommendations related to indications, dosages and duration of thromboprophylaxis.^26^ There is low level of evidence and most guidance derive from retrospective studies, prospective cohorts and the opinion of experts.

No evidence supports the use of routine prophylaxis,^27^ therefore the use of risk stratification systems may guide decision-making.^28^ Despite the lack of randomized and controlled studies to guide prevention strategies, there is no doubt that there are benefits to thromboprophylaxis in the reduction of the recurrence of the VTE, and some authors encourage institutions to adopt their own protocols. The clinical choice should be made through shared decision-making and incorporating patient preferences and values.

## Heparins

Heparins enhance the action of antithrombin – an endogenous anticoagulant. Unfractionated heparin (UFH) and low molecular-weight heparins (LMWHs) do not cross the placental barrier and are not secreted in the maternal milk – thus, are considered safe during pregnancy and the puerperium.^29^ It is important to remember the physiological changes that occur during pregnancy, such as increase of 1) maternal plasmatic volume (40-50%), 2) proteins that reduce the bioavailability of heparin and 3) glomerular filtration rate, increasing renal clearance.^8^ Low molecular-weight heparins (dalteparin and enoxaparin) are the pre- and postnatal drugs of choice for thromboprophylaxis^8^ ^12^ ^14^ ^17^ Studies of the general population demonstrate that LMWHs are as efficient as UFH for thromboprophylaxis as well as safer – osteoporosis, fractures, and the risks of thrombocytopenia induced by heparin are significantly lower with LMWHs.^8^ ^13^ Neither UFH or LMWHs are associated with significant bone demineralization when used in prophylactic dosages during the period of pregnancy.^30^ Both drugs show a clinically significant incidence of bone loss (≥ 10%) in only between 2 and 2.5% of patients.^31^ The potential advantages of LMWHs for short- and long-term use include predictable treatment response, longer half-lives, less allergic reactions, and lower risk of thrombocytopenia induced by heparin.^8^

Women receiving prophylactic dosages of LMWHs during pregnancy must be counseled to discontinue its use 24 hours before scheduled childbirth or as soon as they begin labor (contractions, vaginal bleeding, rupture of membranes or loss of mucus plug).^22^ Hematoma in the vertebral canal is a rare complication of the neuraxial blockade. It is recommended to wait at least 12 hours between the last prophylactic dose of LMWHs (low dosage) and the blockade.^32^ Protocols for LMWHs dosages are detailed in Tables 1 and 2.

**Table 2:** Thromboprophylaxis during pregnancy with low molecular-weight heparin according to the American College of Obstetricians and Gynecologists (extracted from ACOG)^8^

Regimen	Dosage
Prophylactic dosage	Enoxaparin 40 mg SC once a day Dalteparin 5,000 U SC once a day
Intermediate dosage	Enoxaparin 40 mg SC 12/12h Dalteparin 5,000 U SC 12/12h
Adjusted dosage (therapeutic)	Enoxaparin 1mg/Kg 12/12h Dalteparin 200 U/Kg once a day

Abbreviation: SC, subcutaneous.

**Source:** ACOG Practice Bulletin No. 196.^8^

The ideal dosage of LMWHs for thromboprophylaxis during pregnancy is unknown and will possibly be clarified after the publication of the results of the Highlow Trial (NCT 01828697; www.highlowstudy.org), a randomized and multicenter study being done that intends to comparatively assess the safety and effectiveness of prophylactic versus intermediate dosages of LMWHs.^33^

It should be observed that robust data is not available to support the use of a specific regimen by pregnant or puerperal obese women, therefore, in practice, the recommendations of the RCOG^13^ are followed. A single randomized clinical trial was published with data on thromboprophylaxis in the population of obese pregnant women undergoing cesarean delivery, showing that when compared to the fixed daily dose (40mg/day), the weight-based enoxaparin dose administered twice daily (0.5mg/ kg 12/12h) more effectively achieved prophylactic levels of anti-Xa activity without reaching therapeutic levels.^19^ Routine platelet monitoring of patients using LMWHs is not necessary, unless there is a history of exposure to UFH.

Regarding anti-Xa activity, no routine dosage indication is established in the main guidelines. Those advocating against routine monitoring emphasize that the correlation of anti-Xa activity with gestational outcome and recurrence of VTE is scantly understood.^13^ ^34^ However, a retrospective study has reported that 79% of the patients receiving prophylactic LMWHs were outside the target anti-Xa activity range (0.2-0.6 UI/mL), with a recommended dosage adjustment when < 0.3 UI/mL.^34^ During the peripartum of high-risk patients, LMWHs could be replaced with UFH, considering its shorter half-life, quick monitoring of its effect (aPTT) and easy reversibility.^8^ ^13^ The UFH dosage regimen recommended by The American College of Obstetricians and Gynecologists is described in Table 3.

**Table 3:** Unfractionated heparin dosages according to the American College of Obstetricians and Gynecologists (extracted from ACOG)^8^

Regimen	Dosage
Prophylactic UFH	5,000-7,500 U SC 12/12h in the 1^st^ trimester 7,500-10,000 U SC 12/12h in the 2^nd^ trimester 10,000 U 12/12h in the 3^rd^ trimester
Adjusted dosage (through aPTT) of UFH	10,000 U or more, SC 12/12h – adjusted for aPTT between 1.5-2.5x control 6 hours after injection

Abbreviations: UFH, unfractioned heparin; SC, subcutaneous; aPTT, activated partial thromboplastin time.

**Source:** ACOG Practice Bulletin No. 196.^8^

## Fondaparinux

Fondaparinux is a pentasaccharide that crosses the placental barrier, yet as of today it is not possible to exclude the possibility of damage to the fetus. There are reports of isolated cases of its use in more advanced stages of pregnancy. Its use during this period should be restricted to patients with contraindication to heparin (thrombocytopenia induced by heparin or allergies). In the breastfeeding phase, it is considered safe.^17^

## Vitamin K Antagonists 

Warfarin has always been used for secondary prophylaxis in the nongestational population.^26^ However, findings that vitamin K antagonists (VKAs) cross the placental barrier and cause fetal abnormalities (nasal and member hypoplasia, chondral calcification, scoliosis, fetus intracranial hemorrhage and schizencephaly – clefts in the cerebral hemisphere) from the 6^th^ week of pregnancy, led to a restriction of its use. Besides that, the exposure to VKAs in the 3^rd^ trimester was associated with peripartum fetal hemorrhage, and its use in the 2^nd^ trimester is associated with neurological impairment (cognitive and behavioral).^35^ Despite the risks of VKA use throughout pregnancy, its use is safe during breastfeeding.^8^ ^13^ ^17^ ^30^

## Direct Oral Anticoagulants 

Due to lack of data regarding the effectiveness and safety of direct oral anticoagulants (DOACs) during pregnancy and breastfeeding, its use is not recommended during this period.^31^

## Thromboprophylaxis Strategies during Pregnancy

**Fig. 2:**
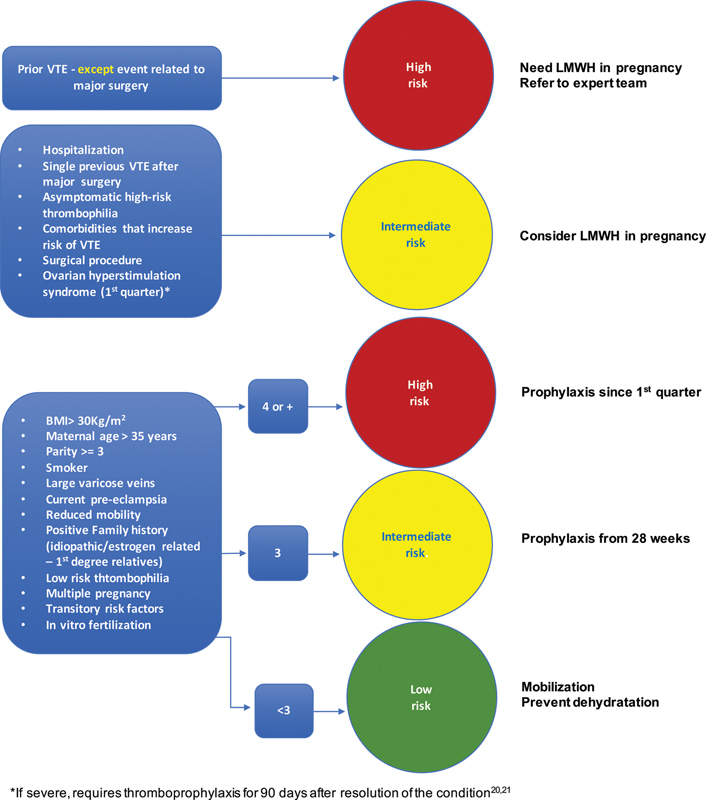
Stratification of the risks during pregnancy (adapted from the Royal College of Obstetricians and Gynaecologists).^13^ **Source:** Reducing the risk of venous thrombosis and embolism during pregnancy and the puerperium.^13^

**Fig. 3:**
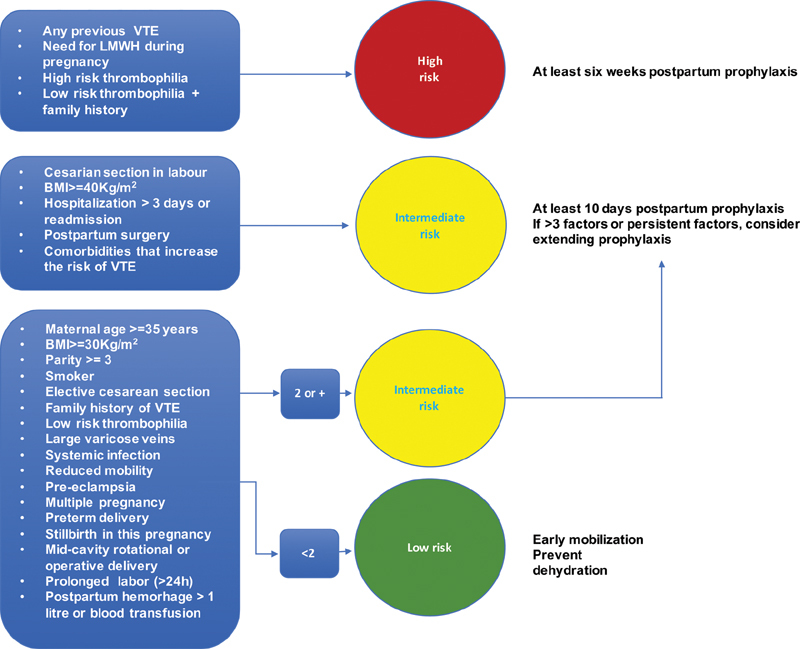
Stratification of the risks during the puerperium (adapted from the Royal College of Obstetricians and Gynaecologists).^13^

There are no large-scale randomized studies, and the main recommendations come from medical guidelines. Following are compiled recommendations from the RANZCOG, the ACCP, the SOGC, the RCOG, the ACOG and the ASH.^8^ ^13^ ^14^ ^15^ ^16^ ^17^ ^18^ The strategy recommended by the RCOG^13^ is considered an achievement in thromboprophylaxis during the pregnancy-puerperal cycle as its implementation at the beginning of this century decreased the maternal mortality rate by > 50% (1.94 maternal deaths for every 100,000 births in 2003–2005 to **0.79** in 2006–2008).^36^ ^37^ The British guideline recommends pharmacological prophylaxis with greater frequency than the North American guidelines. Due to its impact in maternal morbimortality, its recommendations were adopted by other countries as well as by the Safe Motherhood Initiative. A summary of the RCOG^13^ recommendations to prevent thrombosis during pregnancy and the puerperium is demonstrated in Figures 2 and 3.

The ACCP^17^ recommendations include more frequent pharmacological prophylaxis, also due to indications after cesarean sections, based on risk factors. Blondon et al^38^ published a meta-analysis that showed cesarean section as an independent risk factor for VTE postpartum, with greater risks related to emergency procedures. The ASH^14^ recommendation addresses more specific situations concerning thrombophilia, which may be useful for those interested in this subject matter. It is important to emphasize that the lack of thorough studies justifies the variance of conduct between medical societies. For further details, see Box 2.

**Box 2:** Compilation of the ACOG, ACCP, ASH, RANZCOG and SOGC guidelines^8^ ^14^ ^15^ ^16^ ^17^ ^18^

	ACOG	ACCP	ASH	RANZCOG	SOGC
Previous idiopathic VTE **or** associated to hormonal risk factor (estrogen)	Pharmacological prophylaxis during pregnancy and puerperium	Pharmacological prophylaxis during pregnancy and puerperium	Pharmacological prophylaxis during pregnancy and puerperium	Pharmacological prophylaxis during pregnancy and puerperium	Pharmacological prophylaxis during pregnancy and puerperium
Sole prior occurrence of VTE associated to a greater reversible risk factor (non-hormonal)/ without thrombophilia	Pregnancy: Monitoring Puerperium: Pharmacological prophylaxis in the case of additional risk factors (i.e. family history, caesarian section, etc.)	Pregnancy: Monitoring Puerperium: Pharmacological prophylaxis	Pregnancy: Monitoring Puerperium: Pharmacological prophylaxis	Pregnancy: Monitoring Puerperium: Pharmacological prophylaxis	
Background of multiple thrombotic events	Pharmacological prophylaxis during pregnancy and puerperium	Pharmacological prophylaxis during pregnancy and puerperium		Pharmacological prophylaxis during pregnancy and puerperium	Pharmacological prophylaxis during pregnancy and puerperium
Low risk asymptomatic thrombophilia*^1^ **and** negative family history for VTE	Monitoring during pregnancy and puerperium. Prophylaxis in puerperium if additional risk factors.	Monitoring during pregnancy and puerperium	Monitoring during pregnancy and puerperium		
Low risk asymptomatic thrombophilia **and** positive family history for VTE	Monitoring or prophylaxis during pregnancy and pharmacological prophylaxis during puerperium	Monitoring during pregnancy and pharmacological prophylaxis during puerperium	If C and S deficiency does not indicate primary prophylaxis during pregnancy, only in the puerperium. If heterozygosis for FVL or mutant prothrombin does not indicate it.	Pregnancy: observation unless other risk factors Puerperium: consider prophylaxis especially if other risk factors	
Low risk thrombophilia **and** sole prior occurrence of VTE (anticoagulation already concluded)	Prophylaxis during pregnancy and puerperium	Prophylaxis during pregnancy and puerperium			Prophylaxis during pregnancy and puerperium
High risk asymptomatic thrombophilia*^2^ **and** negative family history for VTE*^3^	Prophylaxis during pregnancy and puerperium	Pregnancy: Monitoring Puerperium: LMWH prophylactic or therapeutic dosage or VKA (RNI:2-3) for 6 weeks	AT deficiency: monitoring during pregnancy and puerperium. Mutant prothrombin or homozygous V Leiden factor: monitoring in pregnancy and prophylaxis during puerperium		Pregnancy and puerperium: prophylaxis if any high-risk thrombophilia
Patient receiving anticoagulants and becomes pregnant	Pregnancy: adjusted dosage of LMWH or UFH Puerperium: therapeutic anticoagulation (VKA and LMWH may be used during breastfeeding)	Pregnancy: LMWH in therapeutic dosage or 75% of the dosage Puerperium: therapeutic anticoagulation (VKA and LMWH may be used during breastfeeding)	Pregnancy: LMWH in one dosage or twice a day Puerperium: UFH, LMWH, fondaparinux*^4^, warfarin or acenocoumarol are considered options by ASH (strong recommendation with very low level of evidence)	Pregnancy: therapeutic anticoagulation Puerperium: return to pre-pregnancy anticoagulation	
Dosages of LMWH	Prophylactic, intermediate or adjusted dosage during pregnancy and puerperium	Prophylactic or intermediate dosage during pregnancy and puerperium	Pregnancy: ASH is in favor of the standard dosage and against the use of intermediate dosage Puerperium: standard or intermediate dosage	Prophylactic, intermediate or adjusted dosage during pregnancy and puerperium	Prophylactic, intermediate or adjusted dosage during pregnancy and puerperium

Abbreviations: ACCP, American College of Chest Physicians; ACOG, American College of Obstetricians and Gynaecologists; ASH, American Society of Hematologists; LMWH, low molecular-weight heparin; RANZCOG, Royal Australian and New Zealand College of Obstetricians and Gynaecologists; SOGC, Society of Obstetricians and Gynaecologists of Canada; UFH, unfractionated heparin; VKA, vitamin K antagonists; VTE, venous tromboembolia; INR, International Normalized Ratio; FVL, Factor V Leiden; AT, Antithrombin.

*^1^ Low risk thrombophilia: deficiency in protein S or C, heterozygosis for Factor V Leiden (FVL) or mutant prothrombin G20210A; *^2^ High risk thrombophilia: homozygosis for Factor V Leiden or for mutant prothrombin G20210A, heterozygosis for Factor V Leiden and mutantrothrombin (combined thrombophilia) or deficiency in antithrombin; *^3^ Family history: immediate family members with prior history of thromboembolism; *^4^ The ACCP does not recommend the use of fondaparinux during breastfeeding (degree of recommendation 2C).

## Peripartum Management

To date, there are no randomized controlled trials or systematic reviews that have simultaneously evaluated VTE recurrence outcomes versus hemorrhagic complications, in the context of the management of peripartum anticoagulation, but there are some options for anticoagulation in this context. One strategy is to stop heparinization before induction of scheduled vaginal or cesarean delivery. The suspension time will depend on the type and dose of heparin in use, respecting the deadlines recommended and described in different anesthesiology guidelines.^1^ ^36^

It is recommended that therapeutic LMWHs should be discontinued 24 hours before neuraxial blockade and, in the case of prophylactic dose, 10 to 12 hours before,^32^ ^39^ considering the 24-hour interval increase in some situations.^39^

There is insufficient scientific evidence to recommend a specific 12 to 24 hour interval for patients on intermediate doses. Unfractionated heparin, when administered intravenously, may be suspended 4 to 6 hours before anesthetic blockade.^32^ ^39^ For cases in which UFH is administered subcutaneously, the Society for Obstetric Anesthesia and Perinatology (SOAP)^32^ recommends 12 hours of interval between the use of UFH and neuraxial blockade, if the dose is from 7,500 IU to 10,000 IU.

It is also recommended that regimens with doses above the prophylactic dose (UFH, 5000UI, 8/8h, subcutaneous route) have their coagulation status assessed by activated partial thromboplastin time (aPTT) dosage and/or anti-Xa activity. Additionally, the Brazilian Society of Anesthesiology (SBA, in the Portuguese acronym) additionally recommends the assessment of platelet counts in patients using UFH if it has not been done within 5 days before delivery.^39^

Another strategy is to transition the anticoagulation of LMWHs to UFH, with discontinuation immediately after the onset of labor.

The ASH^14^ has as an additional option to wait for the spontaneous start of labor if the pregnant woman is using prophylactic UFH or LMWHs, reserving the scheduled delivery only for situations in which the pregnant woman is using therapeutic doses.

In face of these possibilities, a multidisciplinary management is recommended, as well as discussing options with the patient so that she may participate in the decision-making process and become aware of the potential impact upon labor analgesia access. This should be done in such a manner that the choices may be individualized, aiming to respect not only maternal preferences but also the safety of the mother-baby binomial.

It is also recommended to evaluate the potential risk factors for postpartum hemorrhage (PPH) in order to identify the most vulnerable patients. The RCOG, in its PPH guideline, mentions the 4Ts associated with increased risk of bleeding: tone (multiple pregnancy, previous PPH, fetal macrosomia, and general anesthesia are associated with uterine hypotonia); thrombin (pre-eclampsia); trauma (episiotomy, perineal laceration) and tissue (accretism or placental retention).^40^

As the primary mechanism of postpartum hemostasis is compression of the uterine vessels by sustained contraction of myometrial fibers, it is assumed that heparinization does not increase bleeding by uterine hypotonia. However, bleeding due to vaginal or cesarean section trauma may be magnified by the use of heparin in the hours before delivery. Thus, the active participation of the obstetric team in the third stage of labor is necessary to minimize trauma and stimulate uterine contractility (through the use of uterotonics such as oxytocin, for example) in women with reported use of heparin in the moments before delivery.^17^

The presence of hereditary thrombophilia does not change the usual obstetric indications that define the mode of delivery. However, induction of full-term vaginal delivery may be considered to better adjust the last dose of anticoagulant, considering the possibility of neuraxial blockade.^8^ ^15^ Scheduling delivery of the patient at high thrombotic risk may exclude an element of uncertainty for the pregnant women as well as for the medical team, thus reducing the likelihood of maternal and fetal exposure to general anesthesia if the patient progresses to emergency cesarean section.

The use of an intrapartum pneumatic compression device should be considered in patients with known thrombophilia until they resume ambulation,^8^ ^15^ and it is important to emphasize that they should not be used in patients with acute thrombotic events due to the risk of embolization of these thrombi. The ACOG recommends that all women with thrombophilia undergoing cesarean section receive at least an intermittent pneumatic compression device.^8^ It is suggested to consider the criteria in Box 2 to determine the appropriate prophylaxis strategy.

## Conclusion

Despite expansive knowledge concerning the risk factors for treatment and prevention of VTE during pregnancy and the puerperal period, management of these patients is extremely difficult due to the potential for complications, along with the need to balance the well-being of mother and fetus during decision-making. Accordingly, there is a general understanding that all women should have the opportunity to take part in choosing their prophylactic and/or therapeutic strategy. Despite the recommendations of guidelines becoming increasingly consistent about the safe and effective use of anticoagulants in order to prevent and treat VTE in this population, as of today there are still significant voids, therefore high-quality research in this area should be a priority. Further information should be collected on issues such as the ideal LMWHs prophylaxis dosage for prevention of recurrent VTE during the pre- and postpartum periods; the absolute risk of VTE during pregnancy, delivery and puerperium in combination with clinical risk factors; and the impact of the application of risk scores to prevent VTE and bleeding risks.

**Box 1:** Selected studies and characteristics

Authors/year	Country	Study design	Main findings
Bates et al (2018)^14^–American Society of Hematology	USA	Guideline	The panel agreed on 31 recommendations related to the treatment of VTE and superficial vein thrombosis, diagnosis of VTE, and thrombosis prophylaxis
American College of Obstetricians and Gynecologists (2018)^15^	USA	Guideline	Summarizes the available data to provide practical approaches
Stephenson et al (2016)^19^	USA	Randomized controlled trial	Comparing two enoxaparin dosing strategies at achieving prophylactic anti-Xa levels in women with a body mass index (BMI) ⩾ 35 (kg/m2) postcesarean delivery, the authors found that weight-based dosing twice daily more effectively achieved prophylactic anti-Xa levels than fixed dosing daily
RCOG (2015)^13^	UK	Guideline	Summarizes the available data and the quality of the evidence to provide practical approaches
Chan et al (2014)^16^–SOGC	Canada	Guideline	Summarizes the available data and the quality of the evidence to provide practical approaches
Bates et al (2012)^17^–ACCP	USA	Guideline	Summarizes the available data to provide practical approaches
McLintock et al (2012)^18^ - RANZCOG	Australia/ New Zealand	Guideline	Compilation of recommendations from the Specialty Society Guidelines

Abbreviations: ACCP, American College of Chest Physicians; RANZCOG, Royal Australian and New Zealand College of Obstetricians and Gynaecologists; SOGC, Society of Obstetricians and Gynaecologists of Canada; VTE, venous thromboembolism;
